# Supplementary material for: Bedside Risk Scoring for Carbapenem-Resistant Gram-Negative Bacterial Infections in Patients with Hematological Malignancies
Source: Infect Dis Rep. 2025 Aug 1;17(4):92. doi: 10.3390/idr17040092 (PMC12385919; doi:10.3390/idr17040092)
Supplement: Supplementary file 1 [file idr-17-00092-s001.zip › idr-3714496-supplementary.pdf]

Supplementary Table S1: Prednisone equivalent doses

S	Glucocorticoid Effect	Mineralocorticoid Effect
Hydrocortisone (20 mg)	1	1
Prednisone (5 mg)	4	0.7
Methylprednisolone (4 mg)	5	0.5
Dexamethasone (0.5 mg)	30	0
Betamethasone (0.6 mg)	25-50	0

Table S2. Evaluation of the performance of the models created for the risk score of carbapenem resistance in hematological malignancy patients with carbapenem-resistant and susceptible GNBIs and diagnostic statistics for different cut-off values.

Model	Overall Performance of Models		Cutoff Value and Diagnostic Statistics				
	AUC (%95 CI)	*p*	Cutoff Value	Sensitivity (%95 CI)	Selectivity (%95 CI)	PPV(%95 CI)	NPV(%95 CI)
**Model-1**	.830(.762-.898)	<0.001	>0.5	.938(.848-.983)	.322(.228-429)	.496(.404-.588)	.879(.718-.966)
			>1.0	.938(.848-.983)	.367(.268-.475)	.513(.419-.606)	.892(.746-.970)
			>1.5	.766(.643-.863)	.800(.703-.877)	.731(.609-.832)	.828(.732-.900)
			>2.0	.750(.626-.850)	.822(.727-.895)	.750(.626-.850)	.822(.727-.895)
			>2.5	.563(.433-.686)	.911(.832-.961)	.818(.673-.918)	.746(.654-.824)
			>3.0	.563(.433-.686)	.911(.832-.961)	.818(.673-.918)	.746(.654-.824)
			>3.5	.438(.314-.567)	.944(.875-.982)	.849(.610-.949)	.703(.613-.782)
			>4.0	.203(.113-.322)	.989(.940-1.000)	.929(.661-.998)	.636(.550-.715)
			>4.5	.203(.113-.322)	.989(.940-1.000)	.929(.661-.998)	.636(.550-.715)
			>5.0	-	-	-	-
			>5.5	-	-	-	-
**Model-2**	.826(.759-.893)	<0.001	>0.5	.984(.916-1.000)	.211(.132-.310)	.470(.383-.558)	.950(.751-1.000)
			>1.0	.859(.750-.934)	.567(.458-.671)	.585(.479-.686)	.850(.734-.929)
			>1.5	.859(.750-.934)	.567(.458-.671)	.585(.479-.686)	.850(.734-.929)
			>2.0	.672(.543-.784)	.867(.779-.929)	.782(.650-.882)	.788(.694-.864)

			>2.5	.641(.511-.757)	.889(.805-.954)	.804(.669-.902)	.777(.684-.853)
			>3.0	.578(.448-.701)	.900(.819-.953)	.804(.661-.906)	.750(.658-.828)
			>3.5	.406(.285-.536)	.944(.875-.982)	.839(.663-.946)	.691(.601-.771)
			>4.0	.406(.285-.536)	.944(.875-.982)	.839(.663-.946)	.691(.601-.771)
			>4.5	.156(.078-.269)	1.000(.960-1.000)	1.000(.692-1.000)	.625(.541-.704)
			>5.0	.156(.078-.269)	1.000(.960-1.000)	1.000(.692-1.000)	.625(.541-.704)
			>5.5	-	-	-	-
**Model-3**	.831(.764-.899)	<0.001	>0.5	.953(.869-.990)	.267(.176-.370)	.480(.391-.571)	.889(.708-.977)
			>1.0	.923(.827-.974)	.367(.268-.475)	.509(.414-.603)	.868(.719-.956)
			>1.5	.828(.713-.911)	.733(.630-.821)	.688(.573-.789)	.857(.759-.927)
			>2.0	.828(.713-.911)	.756(.654-.840)	.707(.590-.806)	.861(.765-.928)
			>2.5	.594(.464-.715)	.889(.805-.945)	.792(.650-.895)	.755(.662-.833)
			>3.0	.594(.464-.715)	.889(.805-.945)	.792(.650-.895)	.755(.662-.833)
			>3.5	.469(.343-.598)	.922(.846-.968)	.811(.648-.920)	.709(.618-.790)
			>4.0	.344(.230-.473)	.956(.890-.988)	.846(.651-.956)	.672(.583-.752)
			>4.5	.313(.202-.441)	.978(.922-.997)	.909(.708-.989)	.667(.579-.746)
			>5.0	.094(.035-.193)	1.000(.960-1.000)	1.000(.541-1.000)	.608(.525-.687)
			>5.5	.094(.035-.193)	1.000(.960-1.000)	1.000(.541-1.000)	.608(.525-.687)
